# Geographical distribution of health indicators related to snake bites and envenomation in Morocco between 1999 and 2013

**DOI:** 10.4178/epih.e2018024

**Published:** 2018-06-16

**Authors:** Faiçal El hattimy, Fouad Chafiq, Hinde Hami, Abdelghani Mokhtari, Abdelmajid Soulaymani, Soulaymani Bencheikh Rachida

**Affiliations:** 1 Laboratory of Genetics and Biometry, Faculty of Science, Ibn Tofail University, Kenitra, Morocco; 2 Moroccan Anti-Poison and Pharmacovigilance Center, Rabat, Morocco; 3 Faculty of Medicine and Pharmacy, Mohammed V University, Rabat, Morocco

**Keywords:** Snake bites, Epidemiology, Morbidity, Mortality, Time-space trends, Morocco

## Abstract

**OBJECTIVES:**

Envenomation from snake bites is a significant cause of morbidity and mortality worldwide. The aim of this study was to describe the epidemiological features of snake bites in Morocco and to evaluate time-space trends in snake bite incidence, the mortality rate, and the case-fatality rate.

**METHODS:**

This is a retrospective study of snake bite cases reported to the Moroccan Poison Control Center between 1999 and 2013.

**RESULTS:**

During the study period, 2,053 people were bitten by snakes in Morocco. Most victims were adults (55.4%). The average age of the patients was 26.48±17.25 years. More than half of the cases (58.1%) were males. Approximately 75% of snake bites happened in rural areas, and 85 deaths were recorded during this period. The incidence of snake bites remained generally steady over the 15-year period of this study, with a marked increase noted since 2012. The mortality rate has increased slightly, from 0.02 deaths per 100,000 inhabitants in 1999 to 0.05 in 2013. The geographical distribution of snake bite cases in the regions of Morocco showed that Tanger-Tétouan had the highest annual incidence of snake bites (1.41 bites per 100,000 inhabitants). However, the highest annual mortality rates were recorded in the Guelmim-Es Semara and Souss-Massa-Drâa regions (0.09 deaths per 100,000 inhabitants for both regions).

**CONCLUSIONS:**

The geographical distribution of the incidence, mortality, and case-fatality rates of snake bites in Morocco showed large disparities across regions during the three 5-year periods included in this study, meaning that certain areas can be considered high-risk for snake bites.

## INTRODUCTION

Snake bite is a neglected public health problem in many tropical and subtropical regions of the world, especially the Middle East, North Africa, South Asia, and Latin America, although the mortality and morbidity rates from snake bite are high [1,2]. Although the exact number of snake bites is unknown, an estimated 5.4 million people are bitten every year, resulting in 1.8 to 2.7 million cases of poisoning. As per the World Health Organisation fact sheet on snake bites, an estimated 81,000 to 138,000 people die each year as a result of snake bites, and there are approximately 3 times more cases of amputations and other definitive disabilities [3].

In Morocco, a total of 1,423 snake bites were reported from 1992 to 2007, with a case-fatality rate of 5.7% [4]. Well aware of the seriousness of the problem, the Moroccan Poison Control Centre (MPPC) has been engaged since 2008 in the fight against snake bites. Moreover, since May 20, 2013, the fight against snake bites has been integrated into the national envenomation control strategy [5]. The aim of this study was to describe the epidemiological characteristics of snake bites and to evaluate time-space trends in snake bite incidence, the mortality rate, and the case-fatality rate in Morocco over a 15-year period from 1999 through 2013.

## MATERIALS AND METHODS

This is a retrospective study of snake bite cases that were reported to the MPCC from a national poisoning surveillance system over a 15-year period from 1999 to 2013. Data were collected from poisoning declaration forms and phone calls received by the Toxicological Information Unit from the public and healthcare professionals.

The MPCC is a national institution with the mandate to provide toxicological information and advice and to oversee the management of poisoning cases in order to reduce morbidity and mortality related to poisoning in Morocco. Its main functions are toxicovigilance activities, which comprise research, education, and training in the prevention and treatment of poisoning [6]. The following data were collected: sex, age, residence, date, location of the incident (urban or rural), region, signs and symptoms, and outcome. The age distribution of bite victims was given according to international standards: toddler, 1-4 years; child, 5-14 years; adolescent, 15-19 years; adult, 20-74 years; and elderly, 75 years or older [7]. Frequencies were calculated to describe the characteristics that were studied. Adjustment to the chi-square statistic was used for testing the equality of proportions of different qualitative variables. The p-values of 0.05 or less were considered to indicate statistical significance [8]. The statistical analysis was conducted using SPSS version 20 (IBM Corp., Armonk, NY, USA). The incidence and mortality rates for snake bites were calculated using the population projections for Morocco produced by the High Commissioner for Planning. We used ArcGIS software (Esri, Redlands, CA, USA) to create maps illustrating the incidence, mortality, and case-fatality rates of snake bites calculated for 16 regions over three 5-year periods starting in 1999.

## RESULTS

During the period 1999-2013, a total of 2,053 cases of snake bites were reported to the MPCC, with an average of 137 cases per year. Table 1 shows the characteristics of snake bites in Morocco between 1999 and 2013. According to the results, 58.1% of the cases were males, with a male-to-female ratio of 1.38 (p < 0.001). The average age of the patients was 26.48 ± 17.25 years. The majority of the bite victims were adults (55.4%), followed by children aged 5-14 years (26.2%). The victims ranged in age from 12 months to 98 years old. As shown in Table 1, more than half of snake bites occurred in public places (57.0%) and about one-third at home (33.6%). Snake bites were most common among people living in rural areas (74.7%), while those recorded in urban areas represented 25.3% of the cases. The majority of bites occurred during the summer and spring seasons (42.9% and 35.9%, respectively), peaking in June (21.0%).

The chi-square test showed significant differences in the case-fatality rate across age categories, as well as between participants living in rural and urban areas.

Of the bite cases, 66% were symptomatic. As shown in Table 2, the gastrointestinal tract was the most frequently affected system (555 cases), followed by heart rate and rhythm (225 cases), the body as a whole (205 cases), the central and peripheral nervous system (178 cases) and the respiratory system (140 cases). The most commonly observed clinical signs were nausea and vomiting (494 cases), local swelling (350 cases), localized pain (240 cases), and tachycardia (214 cases).

The following table shows the incidence, mortality, and case-fatality rates for snake bites by year in Morocco during the period of study. As shown in Table 3, between 1999 and 2013, the average annual incidence was 0.46 per 100,000 inhabitants. The incidence rates generally remained steady over the 15-year study period, with a marked increase noted since 2012 (0.67 bite cases per 100,000 inhabitants). The number of deaths caused by snake bites from 1999 through 2013 was 85, with an average annual mortality rate due to snake bites of 0.02 per 100,000 inhabitants. The mortality rate increased slightly from 0.02 deaths per 100,000 inhabitants in 1999 to 0.05 in 2013. The case-fatality rate for snake bites ranged from 0.8 to 7.5%, representing 1 to 14 deaths per year in Morocco.

**Figure 1.**
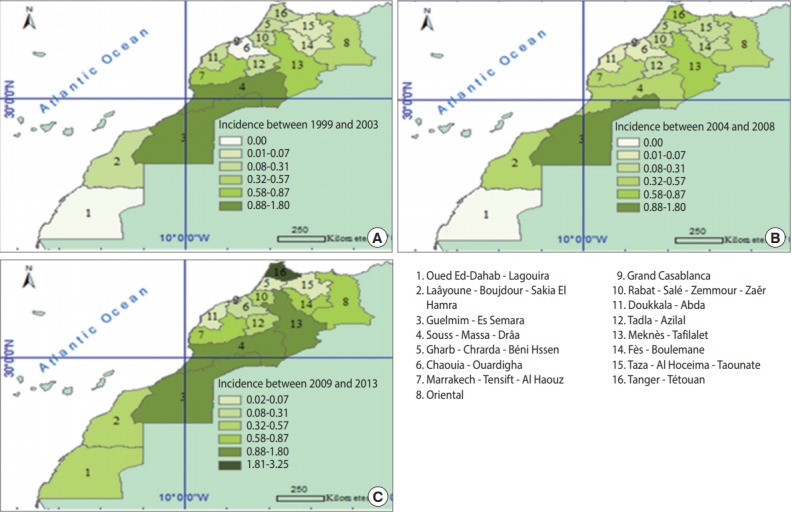
Incidence rates per 100,000 population of snake bites by region over three 5-year periods of (A) 1999-2003, (B) 2004-2008, and (C)2009-2013.

**Figure 2.**
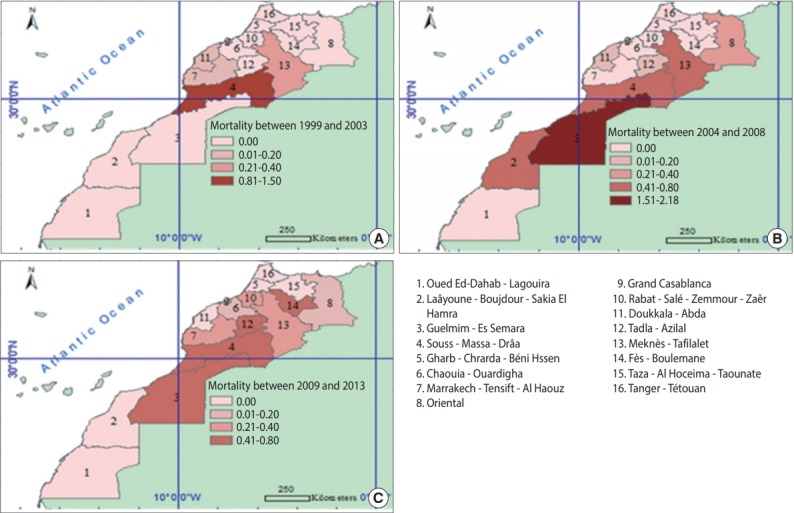
Death rates per 100,000 population for snake bites by region over three 5-year periods of (A) 1999-2003, (B) 2004-2008, and (C) 2009-2013.

Figure 1 shows the incidence of snake bites in the regions of Morocco during the three 5-year periods of 1999-2003, 2004- 2008, and 2009-2013. The highest annual incidence rates of snake bites during the study period were recorded in the Tanger-Tétouan region (1.41 per 100,000 population), followed by Guelmim-Es Semara (1.28 per 100,000 population) and Souss-Massa-Drâa (0.93 per 100,000 population). During the periods 1999-2003 and 2004-2008, as shown in Figure 2, Guelmim-Es Semara had the highest incidence rates of snake bites (1.79 and 1.18 cases per 100,000, respectively), possibly due to low demographics and underreporting in other regions. However, during the period 2009-2013, the highest rate of snake bites was recorded in the Tanger-Tétouan region (3.20 cases per 100,000 population). This high rate could have been associated with the efforts made by health professionals in the Tanger-Tétouan region, increasing levels of awareness of the importance of officially registering snake bites, and the diversity of its ophidian fauna.

Figure 2 shows the mortality rates for snake bites in various regions for the three 5-year periods of 1999-2003, 2004-2008, and 2009-2013. During the study period, Guelmim-Es Semara and Souss-Massa-Drâa showed the highest annual mortality rates for snake bites (0.09 deaths per 100,000 inhabitants in both regions). In 1999-2003, the highest mortality rate for snake bites in Morocco was recorded in the Souss-Massa-Drâa region (1.30 deaths per 100,000 inhabitants), followed by Meknès-Tafilalet (0.30 deaths per 100,000 inhabitants). However, in the second 5-year period from 2004 to 2008, Guelmim-Es Semara showed the highest mortality rate, followed by Laâyoune-Boujdour-Sakia El Hamra (2.20 and 0.80 deaths per 100,000 inhabitants, respectively). Between 2009 and 2013, as shown in Figure 2, Fès-Boulemane had the highest mortality rate for snake bites, at 0.80 deaths per 100,000 inhabitants. These results can be explained by the abundance of venomous species in these 3 regions.

**Figure 3.**
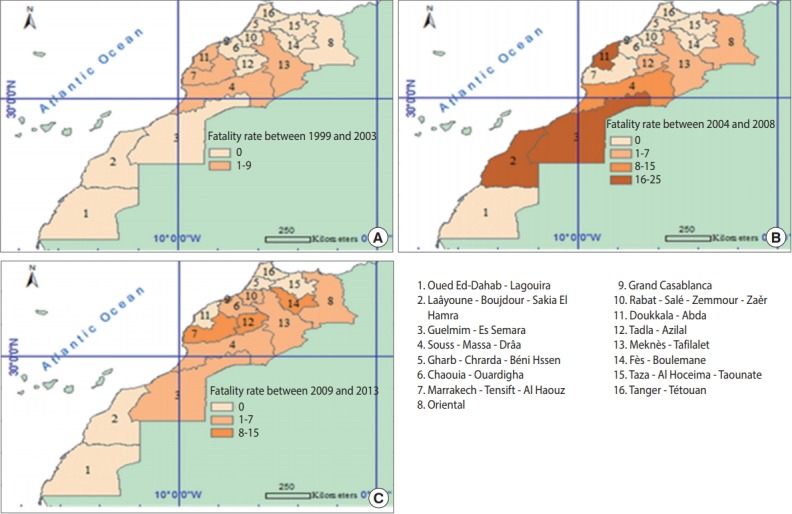
Fatality rates (%) for snake bites by region over 5-year periods of (A) 1999-2003, (B) 2004-2008, and (C) 2009-2013.

During the first 5 years (1999-2003), the highest case-fatality rate for snake bites was recorded in the Souss-Massa-Drâa region (9.0%). However, in the second period ranging from 2004 to 2008, Doukkala-Abda showed the highest case-fatality rate (25%). Between 2009 and 2013, Fès-Boulemane and Marrakech-TensiftAl Haouz had the highest case-fatality rate (13%) (Figure 3).

Finally, a large disparity in incidence rates during the first and third periods of the study was observed, in which some regions had high levels of reported snake bites, such as the Guelmim-Es Samara region and the Souss-Massa-Drâa region, while others had few or no reports of snake bites, such as the Taza-Al Hoceima-Taounate region and the Chaouia-Ouardigha region. In contrast, during the second period, this difference was less striking, except in the Souss-Massa-Drâa region. This disparity may be attributable to variation in the climate and the local agricultural zones across the regions. The mortality and case-fatality rates diminished in the third period, becoming more homogeneous.

## DISCUSSION

Snake bites are an important public health problem in many regions of the world, especially in rural areas. During the 15-year period from 1999 to 2013, 2,053 cases of snake bites were registered in Morocco, 85 of which were fatal. The number of reports has increased considerably since 2012, due to the development of specific snake bite hospitalization records. These records were made available in different regions following the ministerial letter of September 21, 2012, which required regional branches to send copies of said records to the MPCC [9]. In addition to these measures, a national envenomation control strategy (based on 6 axes, including improved case reporting and data collection) was established [9].

Most victims were adults and children under the age of 15 years, as has been shown in previous studies [5]. This can be explained by the age structure of the population of Morocco.

According to the results, most incidents occurred during the warmer spring and summer months, when snakes are more active [10,11]. Snakes are cold-blooded animals and need warm weather to become active hunters. The warm period of the year coincides with the harvesting period: people are more active in the fields and therefore are more frequently exposed to snakes, which explains the high frequency of bites and envenomations outside the home. In our study, most snake bites happened in rural areas, as has been shown in many studies [12,13].

The variability and severity of the observed clinical signs can be explained by several factors: the species responsible for the bite, the size of the snake, its physiological state, the amount of venom inoculated at the site of the bite, and the victim’s health condition [14].

The incidence of snake bites remained relatively steady for more than 15 years, with an annual incidence of 0.46 bites per 100,000 inhabitants. These rates are similar to those found in North Africa by 1.00 Kasturiratne et al. [1] and far lower than those observed in some parts of sub-Saharan Africa, Asia, and Latin America, where rates have been reported to vary from 8 to 130 bites per 100,000 inhabitants [15-17]. The annual mortality rate for snake bites was 0.02 per 100,000 population. This rate is close to those recorded in the Near and Middle East, North Africa, and Latin America, and lower than those recorded in Asian and sub-Saharan African countries [1].

The geographical distribution of snake bite cases across the regions of Morocco during the three 5-year periods of 1999-2003, 2004-2008, and 2009-2013 shows that over the first 5-year period (1999-2003), the Guelmim-Es Semara and Souss-Massa-Drâa regions had the highest rates (1.79 bites per 100,000 inhabitants). Between 2004 and 2008, the highest incidence rate of snake bites was recorded in the Guelmim-Es Semara region (1.18 bites per 100,000 inhabitants). However, in the third period, ranging from 2009 to 2013, the highest rate of snake bites was recorded in the Tanger-Tétouan region (3.20 bites per 100,000 inhabitants). This increase could have been related to surveillance efforts deployed by the MPCC. The fight against snake bites has been integrated into the national envenomation control strategy [5-18].

Regarding the geographical distribution of deaths from snake bites, our results show large disparities in the death rates across different regions over the three 5-year time periods of 1999-2003, 2004-2008, and 2009-2013. This is due to the diversity of snake species found in Morocco, their distribution, and their dangerousness. Previous studies have shown that the most dangerous snake species were located in southern Morocco (*Cerastes cerastes*) and the Rif and the Middle Atlas Mountains (*Vipera latastei*) [19]. These disparities between regions were less important in the third period from 2009 to 2013, which may be explained by the increased quality of first aid responses, the provision of timely and efficient care, and the availability of antivenin for treating snake bite envenomation [20,21].

In conclusion, the geographical distribution of incidence enabled us to identify regions with a high risk of snake bites. These regions need more awareness-raising campaigns and improved training of medical staff on first aid and adequate care of patients with snake bites, in order to contribute to the reduction of morbidity and mortality related to snake bites and envenomation.

This study has some limitations, in particular due to the non-exhaustiveness of the database, which did not contain information regarding the species of snake involved, biological analyses, the treatment given to patients, or the patients’ clinical outcomes.

## Figures and Tables

**Table 1.** Characteristics of snake bites in Morocco, 1999-2013

Characteristics	Total^1^	Outcome^1^			Case-fatality rate (%)	Chi-square
		Death	Recovery	Unknown		
Sex						
Female	842 (41.9)	45 (5.3)	487 (57.8)	310 (36.8)	8.5	
Male	1,166 (58.1)	40 (3.4)	702 (60.2)	424 (36.4)	5.4	0.74
Total	2,008 (100.0)	85 (4.2)	1,189 (59.2)	734 (36.5)		
Age (yr)						
Toddler (1-4)	66 (3.3)	5 (7.8)	40 (66.7)	21 (31.8)	11.1	
Child (5-14)	523 (26.2)	42 (8.0)	307 (58.7)	174 (33.3)	12.0	
Adolescent (15-19)	278 (13.9)	4 (1.4)	161 (57.9)	113 (40.6)	2.4	10.77^*^
Adult (20-74)	1,105 (55.4)	32 (2.9)	655 (59.3)	418 (37.8)	4.7	
Elderly (≥75)	24 (1.2)	1 (4.2)	18 (75.0)	5 (20.8)	5.3	
Total	1,996 (100.0)	84 (4.2)	1,181 (59.2)	731 (36.6)		
Residence						
Home	592 (33.6)	16 (2.7)	359 (60.6)	217 (36.7)	4.3	
Workplace	167 (9.5)	10 (6.0)	116 (69.5)	41 (24.5)	8.0	1.30
Public place	1005 (57.0)	48 (4.8)	591 (58.8)	366 (36.4)	7.5	
Total	1764 (100.0)	74 (2.7)	1,066 (60.4)	624 (36.4)		
Location						
Rural	1,208 (74.7)	59 (4.9)	695 (57.5)	454 (37.6)	7.8	
Urban	409 (25.3)	7 (1.7)	275 (67.2)	127 (31.0)	2.5	9.80^*^
Total	1,617 (100.0)	66 (4.1)	970 (60.0)	581 (35.9)		
Seasons						
Autumn	251 (13.3)	8 (3.2)	151 (60.2)	92 (36.6)	5.0	
Winter	147 (7.8)	8 (5.4)	72 (49.0)	67 (45.6)	10.0	
Spring	678 (35.9)	29 (4.3)	426 (62.8)	223 (32.9)	6.4	2.15
Summer	810 (42.9)	32 (3.9)	444 (54.8)	334 (41.2)	8.6	
Total	1,886 (100.0)	77 (4.1)	1,093 (57.9)	716 (38.0)		

Values are presented as number (%).

1 Among cases for whom information was available.

* p<0.05.

**Table 2.** Number of snake bites cases by system organ class affected

System organ class affected	Cases (n)
Gastrointestinal system	555
Heart rate and rhythm	225
Body as a whole	205
Central and peripheral nervous system	178
Respiratory system	140
Central cardiovascular system	118
Other systems	49
Total	1,470

**Table 3.** Annual incidence and severity of snake bites in Morocco, 1999-2013

Year	No. of cases (n)	No. of deaths (n)	Incidence (per 100,000)	Mortality (per 100,000)	Case-fatality rate (%)
1999	117	7	0.41	0.02	6.0
2000	116	4	0.40	0.01	3.4
2001	92	3	0.32	0.01	3.3
2002	115	4	0.39	0.01	3.5
2003	124	9	0.41	0.03	7.3
2004	107	8	0.36	0.03	7.5
2005	96	4	0.32	0.01	4.2
2006	149	4	0.49	0.02	4.7
2007	98	1	0.32	0.01	2.0
2008	96	4	0.31	0.01	4.2
2009	133	1	0.42	0.00	0.8
2010	141	2	0.44	0.01	2.1
2011	93	2	0.29	0.01	2.2
2012	219	8	0.67	0.03	5.0
2013	357	14	1.09	0.05	4.5
Total	2,053	85	0.46	0.02	4.1
